# Overexpression of G6PD and HSP90 Beta in Mice with Benzene Exposure Revealed by Serum Peptidome Analysis

**DOI:** 10.3390/ijerph120911241

**Published:** 2015-09-10

**Authors:** Juan Zhang, Kehong Tan, Xing Meng, Wenwen Yang, Haiyan Wei, Rongli Sun, Lihong Yin, Yuepu Pu

**Affiliations:** Key Laboratory of Environmental Medicine Engineering of Ministry of Education, School of Public Health, Southeast University, Nanjing 210009, China; E-Mails: kehom123@126.com (K.T.); dzmx925@126.com (X.M.); ywwseu@163.com (W.Y.); why_314614@163.com (H.W.); sunrongli5318@gmail.com (R.S.); lhyin@seu.edu.cn (L.Y.)

**Keywords:** benzene, peptidome, biomarkers, G6PD, HSP90 beta

## Abstract

The small peptides representation of the original proteins are a valuable source of information that can be used as biomarkers involved in toxicity mechanism for chemical exposure. The aim of this study is to investigate serum peptide biomarkers of benzene exposure. C57BL/6 mice were enrolled into control group and benzene groups of 150 and 300 mg/kg/d Serum peptides were identified by mass spectrometry using an assisted laser desorption ionization/time of flight mass spectrometry (MS). Differential peptide spectra were obtained by tandem mass spectrometry and analyzed by searching the International Protein Index using the Sequest program. Forty-one peptide peaks were found in the range of 1000–10,000 Da molecular weight. Among them, seven peaks showed significantly different expression between exposure groups and control group. Two peptide peaks (1231.2 and 1241.8), which showed a two-fold increase in expression, were sequenced and confirmed as glucose 6-phosphate dehydrogenase (G6PD) and heat shock protein 90 Beta (HSP90 Beta), respectively. Furthermore, the expression of the two proteins in liver cells showed the same trend as in serum. In conclusion, G6PD and HSP90 beta might be the candidate serum biomarkers of benzene exposure. It also provided possible clues for the molecular mechanism of benzene-induced oxidative stress.

## 1. Introduction

Benzene is a chemical contaminant that is widespread in industrial and living environments [1,2,3,4]. Long-term and high-dose exposure to benzene can cause a variety of harmful effects on the human body including a reduction in peripheral white blood cells (WBC), bone marrow suppression, and even leukemia [5,6,7,8]. Benzene was identified as a human carcinogen by the International Agency for Research on Cancer (IARC) in 1982 [9].

Potential mechanisms of benzene toxicity are involved in metabolic activation and detoxification of benzene in liver and bone marrow [10]; oxidative damage [11], DNA mutation, chromosome aberrations induced by metabolites of benzene [12,13]. The relative biomarkers were also identified to evaluate exposure to benzene [14], indicate the early effects of benzene-exposure [15] or identify the susceptibility population to benzene toxicity [16]. Recently, a system biology approach has offered an integrated analysis of benzene induced alterations in the transcriptome, proteome, and metabolome. Omics-based studies could help to understand the interaction of benzene and genes, and develop novel biomarkers of exposure, early effect and susceptibility to benzene.

Small serum proteins with a molecular weight of <50 kDa are known as serum peptides. Most of these are fragments of macromolecular proteins degraded by internal proteolytic enzymes. The peptides in the circulation can act as biomarkers representative of the original proteins. These peptides were often ignored in past proteomics studies since they are unstable and have too small a molecular weight, or they were simply regarded as interfering molecules [17,18]. However, more and more evidence suggests that these small peptides are a valuable source of information that can be used for chemical exposure and disease diagnosis [19,20]. Since tissue proteins are too big to penetrate epithelial tissue by passive transport and thus to enter the circulation, any disease-specific proteins cannot be detected by blood tests. In contrast, peptides representing these source proteins can enter the circulation where they can be detected. A peptidomics study concerning the leukemia HLA peptides compared SHLA peptidomics of the bone marrow and peripheral blood from patients with multiple myeloma and acute lymphoblastic leukemia and found that 89% of bone marrow-specific peptides could be detected in the peripheral blood [21]. Serum peptides were also found to have a good specificity for chemical exposure and toxicity [22]. In this study, we applied peptidomics analysis and compared the peptide maps of serum samples from control mice and benzene-exposed mice to identify the Variation of peptides in serum associated with hematopoietic injuries induced by benzene exposure.

## 2. Experimental Section

### 2.1. Animals and Benzene Exposure

Thirty-six 8-week-old C57BL/6 male mice (weighing 18–22 g) were obtained from the laboratory animal center of Nanjing Medical University (Nanjing, China, License Number: SCXK (su) 2002-0031). The mice were acclimatized for 1 week in a specific-pathogen free animal facility prior to administration of test substances. Mice were randomly divided into three groups (12 per group): control group (vehicle, corn oil), benzene group 1 (B1: 150 mg/kg of body weight and benzene group 2 (B2: 300 mg/kg b.w.) and were injected subcutaneously with either corn oil (2.5 µL/g b.w.) or a benzene-corn oil mixture once per day, 5 days per week for 4 consecutive weeks. The animals were maintained in a controlled environment with a 12-h light/12-h dark cycle and temperature and relative humidity were maintained at 24 °C ± 1 °C and 55% ± 10%, respectively. During treatment, the body-weights of the mice were measured every week and their general condition was also recorded.

The protocol of experiments was reviewed and approved by the Research Ethics Committee of the Southeast University (approval number: 20131109). Animals were maintained and experiments were conducted in accordance with the Institutional Animal Care and Use Committee of Southeast University.

### 2.2. Collection and Processing of Serum Samples

At the end of the treatment period, the general condition of each mouse was observed and their weights were recorded. The mice were then anaesthetized and blood samples were collected by excising the eyeballs. Part of each blood sample was added to a tube containing anticoagulants and then used to measure blood parameters such as peripheral red blood cells (RBC), white blood cells (WBC), hemoglobin (Hgb), neutrophils and lymphocytes using an automated blood cell analyzer. Other non-anticoagulated blood samples were placed at room temperature for 30 min, then serum was collected by centrifugation at 1000×g and stored at −80 °C. The mice were killed by cervical dislocation to perform gross anatomy. The general conditions of liver, lung, spleen, thymus and bilateral kidneys were observed by the naked eye. Wet weights of liver, lung, bilateral kidneys, spleen and thymus were measured. Organ coefficients (=organ weight/body weight) were calculated based on the above data.

### 2.3. WCX Fractionation and MALDI-TOF MS Analysis

Plasma samples were thawed in an ice bath for 30 min and centrifuged for 10 min at 1500×g. Then, all peptides were processed with weak cationic exchange magnetic beads (WCX-MB) (Fanxing Tech, Beijing, China) according to standard protocol manuals.

In brief, 10 µL plasma were mixed with 10 µL WCX magnetic beads suspension in a 200 µL polypropylene tube and allowed to stand for 5 min. The tube was then placed in a magnetic bead separator for 2 min, to allow all bound peptides to be adsorbed on one site of the tube. After washing the bound peptides three times in wash buffer, the peptides were eluted from theca beads with 5 µL elution buffer, then prepared for further MS analysis.

Before mass spectrometric detection, 1.5 µL eluted peptide was mixed with 10 µL matrix solution (0.4 g/L alpha-cyano-4-hydroxycinnamic acid in ethanol/acetone 2:1). Then, 1 µL of the mixture was spotted onto an ordinary metal target (Bruker Daltonics), allowed to dry, and then this step was repeated two more times. Finally, the processed samples in the target were analyzed by micro-flex MALDI-TOF MS (Bruker Daltonics) in positive ion linear mode. External calibration of the instrument was performed using a mixture of peptide/protein standards between 1000 and 10,000 Da.

### 2.4. Peptide Identification

Peptide identification was performed using a nano-liquid chromatography-electro spray ionization-tandem mass spectrometer (nano-LC/ESI-mass spectrometry/mass spectrometry) (Michrom Bioresources, Auburn, CA, USA). The peptide solutions were loaded onto a nanoaquity UPLC C18 trap column (5 µm, 180 µm × 20 mm (symmetry)), with a flow rate of 15 L/min. The desalted peptides were then analyzed using anano-acquityC18 analytical column (3.5 µm, 75 µm × 150 mm × (symmetry)) at a flow rate of 400 nL/min.

Selected peptides were further purified by gradient separation with the mobile phases A (5% acetonitrile, 0.1% formic acid) and B (95% acetonitrile, 0.1% formic acid). The MS/MS system was operated in the data-dependent positive ion mode with a full MS scan range from 400 m/z to 2000 m/z. The minimum resolution threshold was 100,000; the strongest ion through the second scan was selected for MS/MS fingerprint searching in the national center for biotechnology information blast database.

The data analysis software Bioworks Browser 3.3.1 SP1 was used for Sequest™ retrieval. The retrieval database was the International Protein Index. Proteins were identified by comparison with peptide maps in the database. Parent ion error was set at 100 ppm and fragment ion error was set at 1 Da. The digested mode was non-digested and the variable modification was methionine oxidation. Retrieval parameters were set as delta cn ≥ 0.10, two charges Xcorr 2.6, three charges Xcorr 3.1 and more than three charges Xcorr 3.5.

### 2.5 Protein Levels of G6PD and HSP90 Beta in the Liver

Protein levels of G6PD and HSP90 beta in the liver of mice treated with benzene or control oil were determined by Western blotting. After mixing with loading buffer, total proteins were boiled for 8 min. G6PD and HSP90 beta were then isolated by SDS-PAGE with a 10% separation gel and 5% spacer gel. Separated proteins were transferred to a PVDF membrane, which was then incubated with 5% nonfat milk overnight at 4 °C. Primary rabbit anti-mouse G6PD (Abcam, Cambridge, MA, USA) and rabbit anti-mouse HSP90 beta (Cell Signaling Technology, Danvers, MA, USA) antibodies were optimized at 1:1000 dilution, and incubated with the membrane overnight at 4 °C. After washing with TBST buffer, the membrane was incubated with the secondary antibody (dilution 1:10,000) (Cell Signaling Technology) for 1 h at room temperature. Signals were visualized using a chemiluminescent substrate by exposure to film. Protein expression levels of G6PD and HSP90 beta were determined relative to the corresponding β-actin expression.

### 2.6. Statistical Analysis

The Anderson-Darling test (PAD) were used to gives information about normal distribution. Multiple comparisons were performed using one-way ANOVA for normal distributed data and Kruskal-Wallis for not normal distributed data. Student’s t-test was used for comparisons between groups. *p*-values < 0.05 were regarded as statistically significant.

## 3. Results and Discussion

### 3.1. General Information

Over the four-week exposure period, the weights of the experimental animals in all groups did not change significantly and there were no significant differences in weight among the groups (*p* > 0.05). The kidney-somatic index was significantly higher in the two exposure groups than in the control group (*p* < 0.05). In contrast, the thymus-somatic index in the two exposure groups was significantly lower in the exposure groups (*p* < 0.05) (Table 1).

**Table 1:** Organ coefficient of benzene exposure groups (100×).

Group	Hepato-Somatic Index	Spleen-Somatic Index	Lung-Somatic Index	Kidney-Somatic Index	Thymus-Somatic Index
Control (n = 12)	5.20 ± 0.25	0.24 ± 0.04	0.51 ± 0.07	1.22 ± 0.07	0.19 ± 0.03
150 mg/kg (n = 12)	5.25 ± 0.40	0.21 ± 0.02	0.56 ± 0.07	1.30 ± 0.09 *	0.15 ± 0.03 *
300 mg/kg (n = 12)	5.26 ± 0.36	0.23 ± 0.08	0.52 ± 0.07	1.31 ± 0.08 *	0.13 ± 0.04 *

* Compared with the control group, *p* < 0.05.

### 3.2. Changes in Routine Blood Parameters after Subacute Benzene Exposure

After subacute benzene exposure, routine blood tests showed that in comparison with the control group, WBC, RBC, lymphocytes and percentages of lymphocytes in the two benzene exposure groups were all significantly lower (*p* < 0.05), while the percentages of neutrophils in the two benzene exposure groups were all significantly higher (*p* < 0.05) and the platelet count was significantly higher in the high-dose exposure group compared with the control group (*p* < 0.05) (Table 2). In addition, both RBC and Hgb in the high-dose exposure group were significantly lower than in the low-dose exposure group while platelet count in the high-dose exposure group was significantly higher than in the low-dose exposure group (*p* < 0.05).

**Table 2:** Routine blood test results of the benzene exposure groups.

Item	Control Group (n = 12)	150 mg/kg Group (n = 12)	300 mg/kg Group (n = 12)
WBC (10^9^/L)	6.61 ± 2.11	3.20 ± 0.78 *	3.07 ± 0.92 *
RBC (10^12^/L)	8.13 ± 0.32	7.44 ± 0.38 *	6.93 ± 0.34 *^, #^
Hgb (g/L)	123.17 ± 6.24	118.75 ± 5.29	109.17 ± 4.99 *^, #^
Plt (10^9^/L)	513.67 ± 96.55	539.83 ± 80.5	642.00 ± 153.19 *^, #^
Neut (10^9^/L)	0.96 ± 0.36	0.69 ± 0.25	0.83 ± 0.45
Neut %	14.48 ± 3.00	21.99 ± 7.61 *	25.68 ± 9.73 *
Lym (10^9^/L)	5.59 ± 1.93	2.50 ± 0.71 *	2.22 ± 0.65 *
Lym %	84.69 ± 3.35	77.62 ± 7.84 *	73.45 ± 9.60 *

* Compared with the control group, *p* < 0.05; ^#^ Compared with low-dose exposure group, *p* < 0.05.

### 3.3 MALDI-TOF Analysis

**Figure 1:**
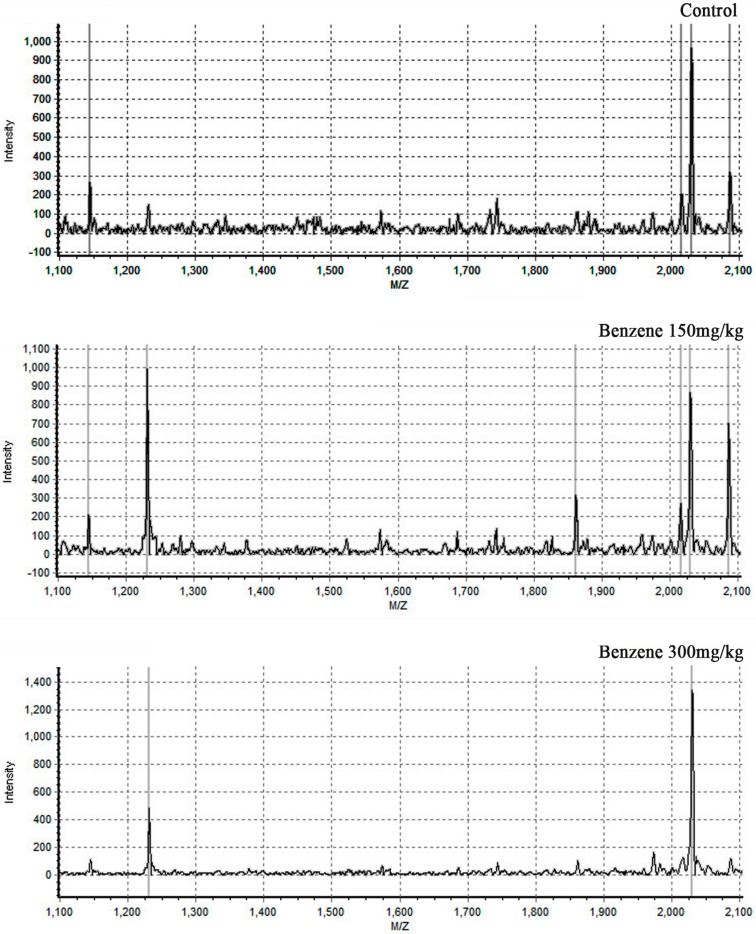
Sum of plasma MALDI-TOF spectra. The mass-to-charge ratio of the mass region of 1100 m/z to 2100 m/z is shown in more detail.

**Figure 2:**
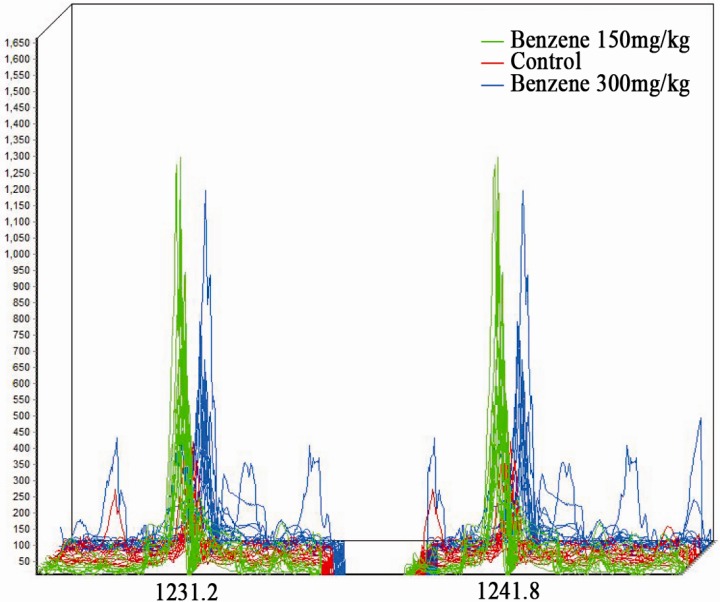
Three-dimensional m/z ratio-intensity maps showing significantly different protein spectra between the three groups. Peptides with molecular weights (MWs) of 1231.2 Da and 1241.8 Da were the two that differed most significantly between control and exposure groups. Green curve, benzene 150 mg/kg; red curve, control group; blue curve, benzene 300 mg/kg.

Serum samples were mixed with a suspension of weak cationic beads. After binding, rinsing, elution and stabilization, serum peptides were extracted from 36 samples from the three groups. Forty-one peptide peaks were identified in the range of 1000–10,000 Da. Peptide spectra over the range of 1100–2100 Da of the three groups are shown in Figure 1. Among the 41 peaks identified, seven peptide peaks exhibited a significant difference between benzene exposure groups and the control group (*p* < 0.05). Four out of the seven peptides were upregulated in the exposure groups (molecular weights were 1231.2, 1241.8, 2796.6 and 2858.4) and the other three were down regulated in the exposure groups (molecular weights were 8204.3, 8224.6 and 8216.1) (Table 3). The peak values of two peptides (1231.2 and 1241.8) were increased more than two-fold in the benzene exposure groups compared to the control group. The MS data were normalized by Flex Analysis software (Brukertech) and cluster analysis was performed on 36 samples based on peptides 1231.2 and 1241.8 using Clinprotool software (Brukertech). As shown in Figure 2, clear differences were evident between control group and exposure groups.

**Table 3:** Peak values of differentially-expressed peptides in benzene exposure groups.

No.	Molecular Weight	Control Group (n = 12)	150 mg/kg Group (n = 12)	300 mg/kg Group (n = 12)	Change
1	1231.2	189.1 ± 118.25	686.08 ± 341.38	383.92 ± 176.12	↑
2	1241.8	48.17 ± 20.59	155.25 ± 46.01	117.75 ± 72.95	↑
3	2796.6	6271.75 ± 4183.03	9372.75 ± 3455.27	10,295.83 ± 2583.29	↑
4	2858.4	119.00 ± 46.28	178.58 ± 39.37	172.00 ± 44.93	↑
5	8204.3	615.00 ± 188.56	372.50 ± 196.53	481.83 ± 149.62	↓
6	8224.6	1295.42 ± 449.83	819.75 ± 383.89	1070.42 ± 443.20	↓
7	8216.1	1299.33 ± 448.98	816.92 ± 380.81	1080.42 ± 442.49	↓

↑ represents upregulated expression in the benzene exposure groups.↓ represents downregulated expression in the benzene exposure groups.

### 3.4. Identification of Peptides

Fragment ion spectra of the two significantly differentially-expressed peptides were obtained by MS/MS. Bioworks Browser 3.3.1 SP1 software was used to perform Sequest™ retrieval from the International Protein Index Database to identify the two peptides. The result indicated that the peptides corresponding to the peaks at 1231.2 and 1241.8 were G6PD and HSP90 Beta, respectively (Table 4).

**Table 4:** Identified peptides.

Molecular Weight	IPI Reference Number	Protein Name	Amino Acid Sequence	Uniprot No.
1231.2 Da	IPI00228385	Glucose-6-phosphate 1-dehydrogenase X	EMVQNLMVLR	Q00612
1241.8 Da	IPI00554929	Heat shock protein 90beta	ADLINNLGTIAK	Q71LX8

### 3.5. Differential Expression of G6PD and HSP90 Beta in Liver Cells

**Figure 3:**
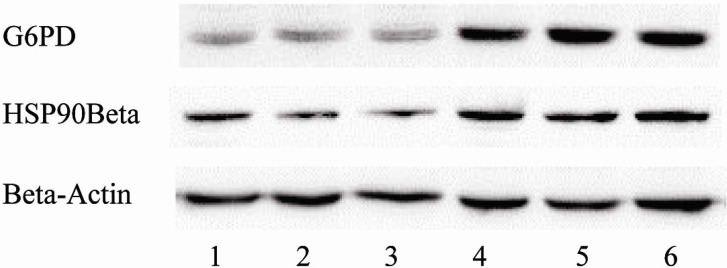
Expression of G6PD and HSP90 Beta in live cells of control mice and benzene-exposed mice; lanes 1, 2 and 3 show extracts from normal control mice and 4, 5, and 6 from 150 mg/kg benzene-exposed mice.

**Figure 4:**
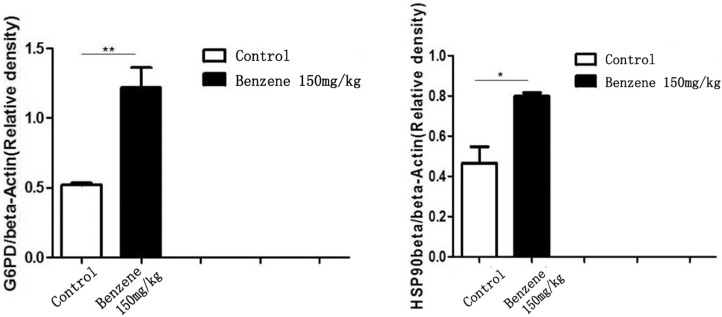
Relative expression (/β-actin) of G6PD and HSP90 Beta in the liver cells of control mice and benzene-exposed mice. ** *p* < 0.01 compared with the control group. * *p* < 0.05 compared with the control group.

The differential expression of G6PD and HSP90 beta in liver cells of control and benzene-exposed mice is shown in Figure 3 and Figure 4. The result revealed that the expression levels of G6PD and HSP90 beta were higher in benzene-exposed mice than in control animals and that the difference was statistically significant.

## 4. Discussion

Long-term exposure to low-dose benzene can cause chronic benzene poisoning including hematopoietic disorders. In this study, we established a subacute mouse model, involving repeated injection of benzene formulation into C57BL/6 mice for four weeks, which can be used to investigate the hematopoietic toxicity of benzene. Routine blood tests showed that the WBC and RBC counts, as well as the percentage of neutrophils and lymphocytes in benzene-exposed mice, all decreased significantly compared with control mice, indicating the obvious hematopoietic toxicity of benzene.

By detecting differentially-expressed serum peptides in benzene-exposed mice which exhibited hematopoietic toxicities and in normal controls, two peptides (m/z 1231.2, 1241.8) were identified. The proteins corresponding to the two peptides were confirmed as G6PD and HSP90 beta by searching the International Protein Index Database. Both proteins were upregulated in benzene-exposed mice. Furthermore, the expression of the two proteins in liver cells showed the same trend as in serum. Significant increases in G6PD and HSP90 beta in benzene-exposed mice implied that these two proteins are likely to participate in the metabolism of benzene and the concomitant stress reaction of the human body and are probably serum biomarkers of benzene toxicity.

G6PD is a housekeeping enzyme present in all cells and tissues [23]. It is the initial as well as the key enzyme in pentose phosphate metabolism bypass and also the first rate-limiting enzyme in the pathway. G6PD catalyzes the conversion of glucose-6-phosphate to glucose-6-phosphate-lactone. During this reaction, NADP+ decreases and NADPH is produced. NADPH is a hydrogen donor for many synthetic reactions in the human body and plays a key role in maintaining the reduced state of glutathione and in protecting cells and cell membranes from damage by oxides [24]. The G6PD deficiency, a mostly asymptomatic inherited red cell abnormality which predisposes to developing favism if the person eats fava beans [25], is a genetic condition associated with decreased activity of the enzyme. The disease can be triggered by various factors such as eating broad beans, taking certain drugs and infection [26,27]. Symptoms of the disease include acute hemolytic anemia and resultant hyperbilirubinemia. Kernicterus can also occur when the disease develops in neonates [26]. Broad beans and some medications have strong oxidizing properties and erythrocytes cannot produce enough glutathione to eliminate excessive ROS, which can cause hemolysis of red blood cells [28]. Recent studies have shown that G6PD deficiency may be involved in the development of diabetes [29], bleeding disorders [30,31] and cancer [32], which are mainly associated with the decrease of antioxidant activity caused by G6PD deficiency. A study conducted by the Diabetes Research Center of Harvard University found that high levels of glucose could inhibit the activity of G6PD in the membrane of endothelial cells, kidney cells, islet β cells, liver cells and red cells, reducing antioxidant activity and increasing oxidative stress and thus inducing cell apoptosis [33]. Oxidative damage is one of the mechanisms by which benzene damages the hematopoietic system and the increase in G6PD is probably a protective mechanism activated in response to the oxidative damage resulting from benzene exposure. G6PD deficiency affects about 400 million people worldwide, and this population might be more susceptible to benzene exposure. The mechanisms underlying susceptibility to benzene are worthy of further study. The susceptible population could thus be identified and protected.

The main function of heat shock proteins (HSPs) is to participate in the folding, assembly, transport and degradation of proteins to regulate the activities and functions of target cells, acting as protective factors when cells are under stress. A member of the HSP family, HSP90, implicated in cell cycling, receptor function, signal transduction, and apoptosis, has two isoforms in the cytoplasm of inducible HSP90α and constitutive HSP90β [34,35]. HSP90 is constitutively expressed at higher levels in cancer. The increase in HSP90 may inhibit tumor cell apoptosis and promote cancer development [36]. Studies have found that HSP expression is closely related to the progression of acute myeloid leukemia (AML). The expression of HSP90 increased in patients with AML and this situation was associated with poor prognosis and resistance to chemotherapy [37]. The induction of HSP90 beta was also found to be protective against oxidative stress [38]. The observed increase of HSP90 beta after benzene exposure in this study is likely to be a protective response, which has the potential to be an early-stage serum biomarker for benzene exposure in a population.

## 5. Conclusions

In this subacute benzene-exposed animal model, differentially-expressed serum peptides were induced in benzene-exposed mice with exhibited hematopoietic toxicities compared with normal controls. Overexpression of G6PD and HSP90 beta might be candidates of serum peptide biomarkers for early exposure to benzene, which is likely to be a protective response to oxidative stress according to the some research results. Their roles in benzene-associated toxicity and G6PD deficiency mechanisms underlying susceptibility to benzene are worthy of further study.
